# Novel approach for the assessment of ovarian follicles infiltration in polymeric electrospun patterned scaffolds

**DOI:** 10.1371/journal.pone.0215985

**Published:** 2019-04-29

**Authors:** Nathalie Raffel, Ralf Dittrich, Tobias Bäuerle, Lisa Seyler, Amir Fattahi, Inge Hoffmann, Aldo Leal-Egaña, Matthias W. Beckmann, Aldo R. Boccaccini, Liliana Liverani

**Affiliations:** 1 Department of Obstetrics and Gynecology, Erlangen University Hospital, Friedrich-Alexander University of Erlangen–Nürnberg, Comprehensive Cancer Center ER-EMN, Erlangen, Germany; 2 Department of Radiology, University Hospital Erlangen, Friedrich-Alexander-University Erlangen-Nürnberg, Erlangen, Germany; 3 Institute of Biomaterials, Department of Materials Science and Engineering, Friedrich-Alexander University of Erlangen-Nürnberg, Erlangen, Germany; Northeastern University, UNITED STATES

## Abstract

Reproductive tissue engineering (REPROTEN) has been recently defined as the application of the tissue engineering approach targeting reproductive organs and several research works are focusing on this novel strategy. Being still an innovative field, most of the scaffold characterization techniques suitable for other tissue targets give inappropriate results, and there is the need to evaluate and investigate novel approaches. In particular the focus of this paper is the evaluation of the infiltration of ovarian follicles inside patterned electrospun scaffolds. Beyond the standard techniques, for the first time the use of magnetic resonance imaging (MRI) for this purpose is proposed and specific protocols for scaffold preparation are reported. Positive results in terms of evaluation of scaffolds incorporating follicles confirm this technique as highly effective for further applications in this field.

## Introduction

Great interest has been recently reported in development of tissue engineering-based approaches for the regeneration of reproductive organs and tissues [1–4]. Considering that these approaches are related to the treatment of infertility in relation to oncological pathologies, they have a relevant impact on the improvement of the patients’ quality of life. In fact, they can be potentially suitable also for pediatric oncological patients, increasing the relevance of focusing research works on this topic [5].

The role of two dimensional and three-dimensional follicle culture systems has been well investigated in the past [6]. In fact, already some biomaterials have been selected for this application, highlighting not only the advantages of using three-dimensional cultures in terms of preservation of the spherical follicles structure but also the pivotal role of biomaterials properties and the related follicles-biomaterial interactions. In this framework biomimetic approaches focussing on the replication of the native healthy tissue morphology and chemistry have been recently developed for the fabrication of artificial ovary prototype [7–9].

In particular, these previous works have focused on the fabrication of gelatin-based scaffolds by 3D printing technique [8] and fibrin clot obtaining fibrillary structures similar to the native human cortex [7]. The authors of the present work also reported the suitability of poly (epsilon caprolactone) (PCL)-based electrospun patterned scaffolds as support for porcine ovarian follicles growth in a previous work [9]. The use of the electrospinning technique is novel for this application and it resulted appropriate because it enables the achievement of fibrillary morphology similar to the native human and porcine cortex. A typical disadvantage of electrospun mats is represented by the limited cell infiltration into the scaffolds due to the high density of the fibers [10]. Several strategies have been proposed in the literature, in particular the combination with other scaffolds fabrication techniques was reported in many studies [11–13]. Besides, the combination with other processes, in order to overcome this limitation, a patterned morphology was proposed [14,15]. In particular, the scaffold exhibits a bimodal distribution of pore size, due to the presence of macro pores with an average size of 300 μm and the fibrous structure of the struts with an average diameter around 1 μm. The assessment of infiltration of porcine ovarian follicles in electrospun patterned scaffolds made of poly (epsilon caprolactone) (PCL) and its blend with gelatin is the focus of the current research work. In particular, several techniques have been investigated and compared in order to assess the advantages of a novel approach based on magnetic resonance imaging (MRI) for this purpose.

The use of MRI for tissue engineering applications has been already reported in literature [16]. In fact MRI was used to assess proteoglycans and collagen synthesis, cellular metabolism, angiogenesis, neuronal activity, mineralization and bone matrix synthesis for application in cartilage, bone, pancreas, cardiac, brain, kidney, liver and ligament/tendon tissue engineering application [17]. An additional application of MRI for tissue engineering is related to the design of custom-printed molds to fabricate anatomically shaped constructs [18]. MRI has not been reported in any application related to women reproductive organs (i.e. ovaries and uterus) tissue engineering (at the moment of the submission of this paper).

## Materials and methods

### Scaffold fabrication

**Fig 1:**
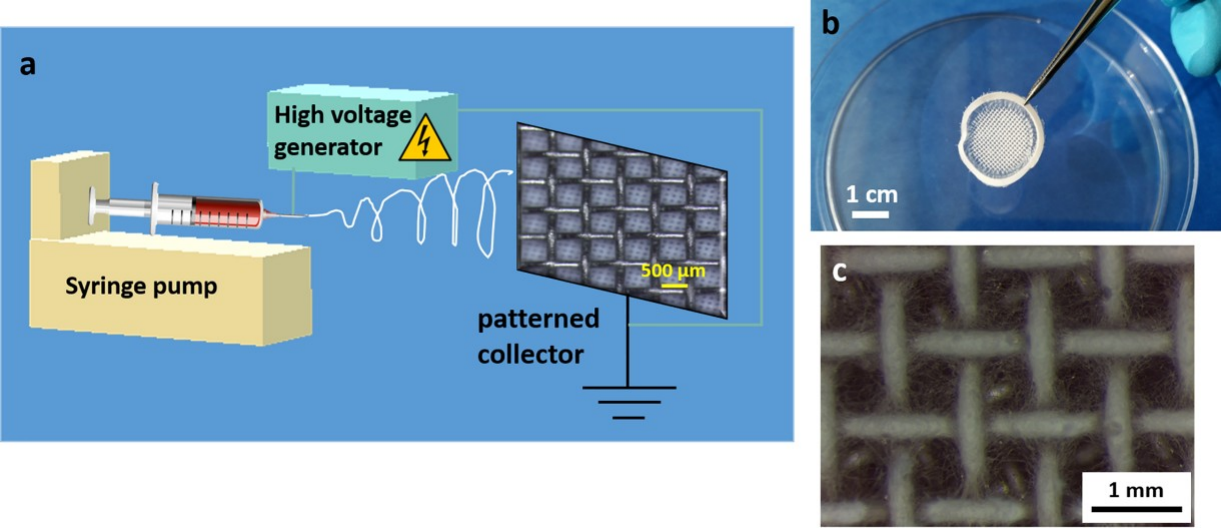
Graphical scheme of (a) the electrospinning process of patterned mats, scale bar of 500 **μ**m reported in the grid, (b) digital camera image of the obtained sample, scale bar of 1 cm, (c) light microscope image of the obtained sample, scale bar 1 mm.

PCL and blended PCL/gelatin patterned electrospun scaffolds were obtained according to the protocol previously reported by the same authors [9,14,15]. Briefly, PCL (80kDa) and gelatin (type A) were purchased from Sigma-Aldrich. PCL was dissolved in glacial acetic acid (20%w/v) while the blend with gelatin was dissolved in a mixture of formic acid and glacial acetic acid (ratio 1:9). The process of electrospinning was optimized for the two different solutions and performed by using a commercial device (EC-CLI, IME Medical Electrospinning) equipped with a gas-shield accessory, which allows the optimization of the instability region and with a climate chamber (set parameter 25°C with 25% relative humidity). The applied voltage was set at 15 kV for both solutions and the distance from the tip (needle of 23G diameter) to the fiber collector was 11 cm. The solutions were extruded with different flow rates: 0.4ml/h for PCL and 0.6ml/h for the blend. A particular collector made of a grid (space between the struts of 500 μm) was used to obtain the patterned mats with macropores. The grid was cut in circular shape (diameter of 2.5 cm) to obtain circular samples suitable to fit the multiwell for cell culture. The electrospinning process, which was run for 5 minutes, is schematically summarized in Fig 1.

### Follicles extraction and seeding

Whole porcine ovaries were collected from the slaughterhouse (registration number DE 09 562 00 38 21; district veterinary office Erlangen-Höchstadt) and washed with Dulbecco’s Phosphate Buffered Saline (DPBS, D8661, Sigma-Aldrich). Half of an ovary was cut into 4–5 mm^3^ pieces by using scalpers and subsequently washed twice with DPBS. Then, tissue pieces were transferred into a 50 ml Falcon tube (Corning Inc.) containing 2.5 ml lysis buffer, which contained 1.5 mg Collagenase (C2674, Sigma-Aldrich) in DPBS (final concentration was 0.6 mg/ml). The tube was incubated in a 37°C water bath for 70 minutes, to enable enzymatic digestion; the tube was gently shaken for about 20 seconds in a 10 minutes interval. Enzymatic reaction was stopped at the end of the incubation time by adding 10 ml of cold DPBS; the tube was then shaken vigorously to detach further follicles from the tissue remains. The content of the tube was transferred into a petri dish (NUNC IVF-Petridishes 90x17mm, Origio) for subsequent follicle collection under an inverted microscope (CKX53, Olympus); used magnification was 250X. Separated follicles were washed 3 times in sterile DPBS and collected in a 20 μl droplet of culture medium before transferring them into the culture well. For each scaffold, a separate droplet in a petri dish (NUNC IVF-Petridishes 60x15mm, Origio) was prepared and the number of collected follicles was checked twice pre-seeding. The electrospun scaffolds were fixed on sample holders (CellCrown 24, Sigma-Aldrich) and put in 24 well plates (Cellstar, Greiner Bio-One). The samples in the 24 well plates were disinfected by UV light irradiation (UV Transilluminator; Bachofer Laboratoriumsgeräte) for 30 minutes before seeding. The number of seeded follicles per sample was 150+/-100; equal numbers of follicles were seeded in the PCL and PCL/gelatin scaffolds as well as in the control samples (0.4 μm PET membrane, Scaffdex), respectively. Experiments were conducted three times for each culture condition (PCL, PCL/gelatin and control), for each experiment the number of seeded follicles was different with respect to the other experiments, but the number of follicles per sample was kept constant. Follicles were pipetted by using a Stripper (Origio) micropipettor with a Stripper Tip (Origio) of 175μm in tip diameter within all collecting and during the seeding steps. The whole droplet with collected follicles was pipetted on the corresponding scaffold during the seeding; both, petri dish and Stripper Tip were checked immediately for remaining follicles, which then were seeded as well. Each scaffold with seeded follicles was cultivated in 1.2 ml of a modified version of the serum free medium used by Telfer *et al* [19]: McCoy’s 5a medium (LifeTechnologies) supplemented with 20 mM HEPES buffer (Gibco), 0.1% BSA (fraction V, CarlRoth), 3 mM L-Glutamine (Gibco), Fungizone (Gibco; final Amphotericin B concentration: 2.5 μg/ml), 0.1 mg/ml of each Penicillin and Streptomycin (Sigma-Aldrich), ITS solution (Sigma-Aldrich; final concentrations: Selenium 4 ng/ml, Transferrin 4.4 μg/ml and Insulin 8 μg/ml), 50 μg/ml Ascorbic acid (Sigma-Aldrich) and 0.272 IE rFSH (Gonal-f, Merck). Whole incubation time for the follicles was 10 days at 38.5°C (porcine body core temperature) in humidified air with 5% CO_2_; half the medium was replaced every second day. Follicles viability was assessed with LIVE/DEAD assay (ThermoFisher Scientific), in which calcein retained from living cells produce green fluorescence and ethidium homodimer-1 interact with nuclear DNA, when cells exhibited damaged membrane, producing red fluorescence. The sample evaluation was performed by using a fluorescence microscope (Axio Scope A1, Zeiss).

### Hematoxylin and Eosin staining (H&E) and light microscopy

The seeded electrospun scaffolds mounted on the scaffold holders (Scaffdex for 24 multiwell plate, Sigma-Aldrich) were immersed in a fixation solution constituted of piperazine-N,N′-bis(2-ethanesulfonic acid) (PIPES), Ethylene glycol-bis(2-aminoethylether)-N,N,N′,N′-tetraacetic acid (EGTA), poly ethylene glycol (PEG) and sodium hydroxide in phosphate-buffered saline (PBS) for 15 minutes. After intensively washing in distilled water, the samples were stained with hematoxylin for 15 minutes. After washing with tap water the samples were immersed for 5 minutes in Scott′s Tap Water (constituted of aqueous solution of sodium bicarbonate and magnesium sulfate). After washing with distilled water the samples were stained with eosin solution (0.4% w/v in aqueous solution made of 60% v/v of ethanol and 5% v/v of acetic acetic) for 5 minutes. The specimens were then washed in ethanol solution of 95%v/v and subsequently in absolute ethanol, before drying them at room temperature for further characterization. All the reagents were purchased from Sigma-Aldrich. The samples were then analyzed with a light microscope (Leica M50, Leica, Germany).

For the investigation of the cross-section the samples were fixed on carbon tape for scanning electron microscopy (SEM), they were then cut with sharp precision scissor and fixed on the cross-section holder for SEM. After being mounted on these holders the samples were analyzed with the light microscope before the investigation at SEM.

### Scanning electron microscopy (SEM)

The morphology of the scaffolds was assessed by using SEM (Zeiss, Germany). The seeded scaffolds (10 days after the seeding) were fixed with a solution constituted by glutaraldehyde, paraformaldehyde, sucrose and sodium cacodylate and dehydrated by using ethanol series. The samples were processed then with the critical point dryer. Afterwards, they were removed from the holder (scaffdex) and fixed on the carbon tape as well as subsequently cut with sharp precision scissor and fixed on the SEM cross-section holder. For all samples the cross section was investigated by using specific cross-section holders for SEM. All the samples were sputtered with gold before the analysis. Also electrospun patterned samples without follicles were fixed on the cross-section holder and used as control. The accelerating voltage used for all the SEM measurements was 1.0 kV.

### Electrospun scaffold fixation and magnetic resonance imaging (MRI)

**Fig 2:**
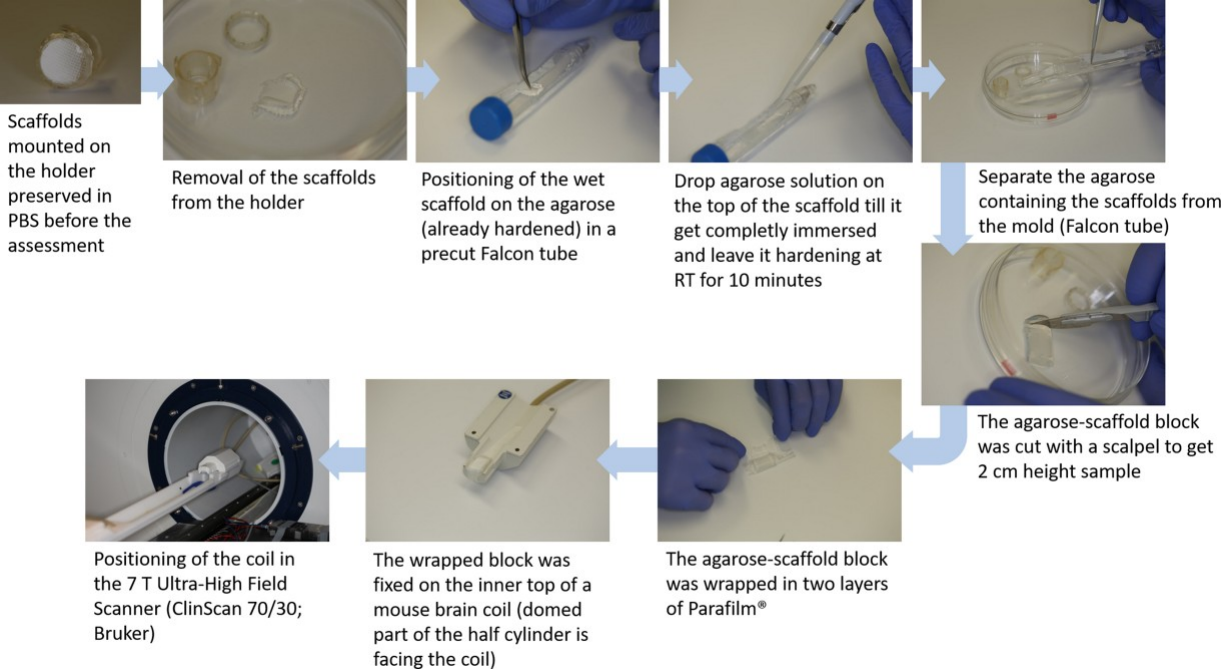
Detailed step-by-step protocol for the sample preparation for MRI assessment by embedding the scaffold in agarose.

To avoid motion artifacts during magnetic resonance imaging (MRI), movement of electrospun scaffolds had to be prevented. The first imaging trials in PBS led to suboptimal images, presumably due to minimal specimen movements during the imaging procedure. Therefore, scaffolds were fixated via agarose embedding for further imaging trials. For the embedding procedure, electrospun scaffolds with follicles were separated from the scaffold holder and fixated in an agarose half cylinder with outer dimensions of 2cm height and a 0.75cm radius. The protocol of the preparation process is reported schematically in Fig 2. Agarose solution was prepared by dissolving 1.5% (w/v) agarose (Sigma-Aldrich, A9539; transition temperature: gel point 36°C (1.5% gel, ±1.5°C)) in deionized water, slightly above the transition temperature. A partly lengthways halved 15ml Polystyrene Conical Tube (Falcon; Corning Inc.) served as cylindrical mold for the agarose basis; lid and end fitting of the tube need to be intact to keep agarose solution within the mold during hardening. About 8ml agarose solution, slightly above the transition temperature, was given into the mold to create the agarose basis; the mass was left 10 minutes at room temperature (RT; 21±1°C) to harden. Then, the wet scaffold was positioned upon the agarose basis by using tweezer; avoiding folding. Subsequently, further agarose solution (about 1.5ml) was dropped via 100–1000μl pipet (Eppendorf Research plus; Eppendorf AG) directly on the scaffold until it was completely surrounded by agarose; the thickness of this additional agarose layer was about 0.25 mm. Air bubbles have to be avoided during agarose embedding, as they would cause artifacts during MRI. After 10 minutes hardening time at RT, the Falcon tube lid was opened to separate the agarose mass from the mold. A scalpel was used to cut down the height of the half cylinder to 2cm, still containing all parts of the scaffold. Embedded scaffolds were wrapped up in two layers of Parafilm M (Pechiney Plastic Packaging) and tape-fixed (Durapore) at a mouse brain coil (^1^H receive-only 2 x 2 mouse brain surface array coil; Bruker) for MRI analysis; the domed part of the half cylinder facing the mouse brain coil.

MRI was performed on a preclinical 7T ultra high field scanner (ClinScan 70/30, Bruker). The imaging-protocol included PD (proton density) weighted turbo spin echo (tse) sequences with the following parameters for agarose embedded samples: PD tse sagittal: repetition time (TR): 2700ms, echo time (TE): 14ms, in plane resolution: 0.025x0.025mm (PCL) and 0.023x0.023mm (PCL/gelatin), matrix: 640, slice thickness: 0.08mm, averages: 2, acquisition time (TA): 12:05h. MRI videos were generated with the image processing tool OsiriX (aycan Digitalsysteme GmbH).

### Confocal microscopy and image analysis

Living cell imaging was used to evaluate the infiltration of the ovarian follicles inside the PCL and PCL/gelatin scaffolds 10 days after seeding by confocal microscopy (Inverted Zeiss Spinning Disc Axio Observer Z1, having an incubation chamber with CO_2_ and controlled temperature. Images were obtained by an EVOLVE 512 EMCCD camera). Z-stack images were obtained, with a distance of 1μm between each picture. The total image had a deepness of 150 μm). In particular, the analysis was performed to the living follicles without fixation. Calcein AM (ThermoFisher Scientific) and Hoechst staining (ThermoFisher Scientific) were used for the evaluation of follicles. In details, Hoechst and Calcein were both added just before the analysis in the concentration of 1μl/mL in cell culture media. ImageJ and its plugin [20], like 3D Viewer were used for the analysis of the confocal images and stacks.

## Results and discussion

**Fig 3:**
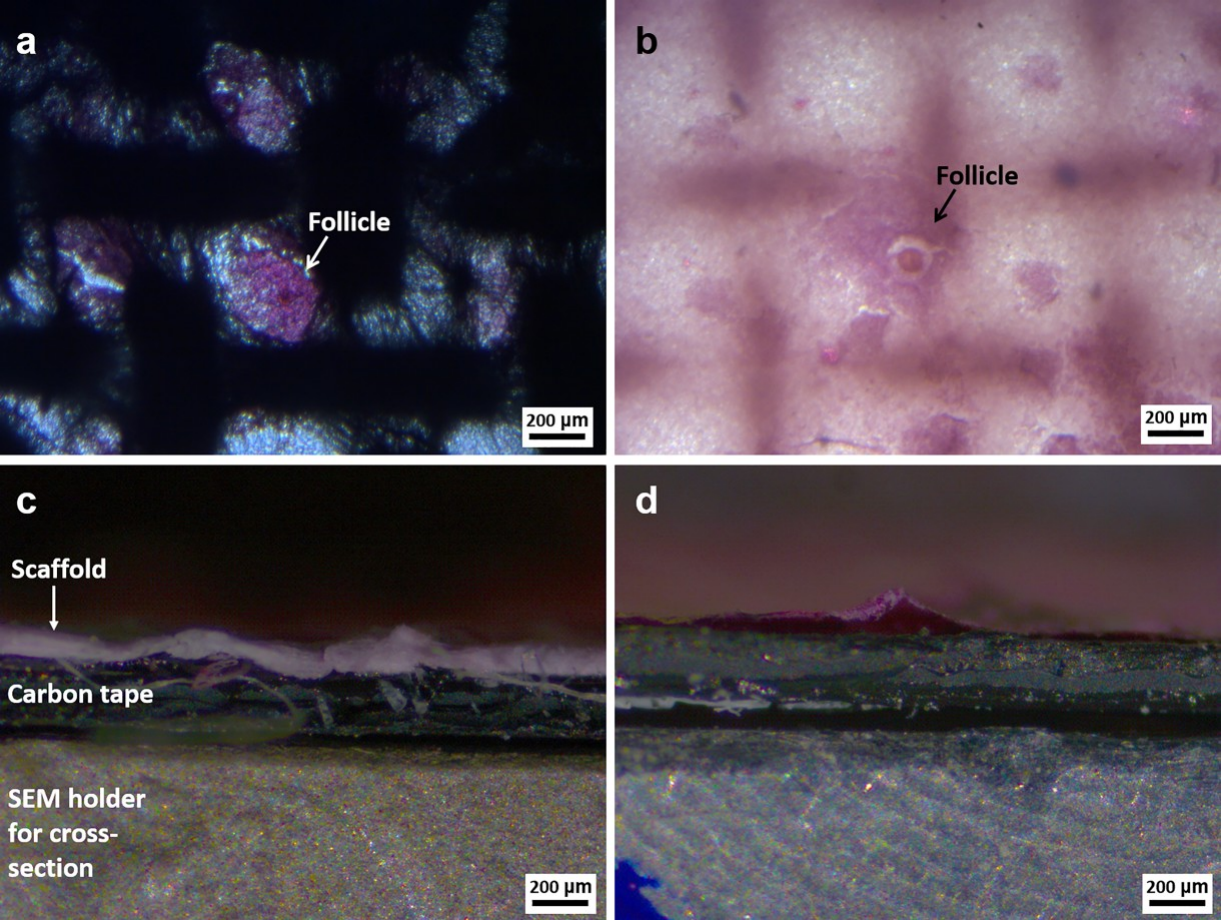
Light microscopy images related to the top view and the cross-section of the patterned electrospun scaffolds seeded with porcine ovarian follicles 10 days after the seeding: (a,c) PCL and (b,d) blend of PCL and gelatin. An indication of the scaffold, carbon tape and holder is reported in c. For all images: magnification 5X, scale bar 200 **μ**m.

Morphological assessment to prove the adhesion and the growth of follicles on the patterned electrospun scaffolds was performed initially with light microscopy. The top view and the cross-section of the samples stained with H&E were analyzed and reported in Fig 3. It is possible to notice that the H&E staining combined with the light microscopy is effective to confirm the presence and the development of the follicles, as clearly shown in Fig 3A and 3B. Respect to previous work based on 3D printed scaffolds [8], it is possible to notice that the follicles adhesion and growth is not just related to the presence of the main struts of the pattern, but the presence of fibers in the middle of the macropores promoted the adhesion of more follicles also in the interior of the pores. From the cross-section analysis reported in Fig 3C and 3D it is not possible to obtain any information regarding the infiltration of the follicles inside the scaffolds. It is only possible to observe the scaffold, which is characterized by the white and pink color (due to H&E staining). Therefore we can conclude that this technique (H&E staining and light microscope analysis) is not suitable for the evaluation of follicles infiltration into scaffolds.

**Fig 4:**
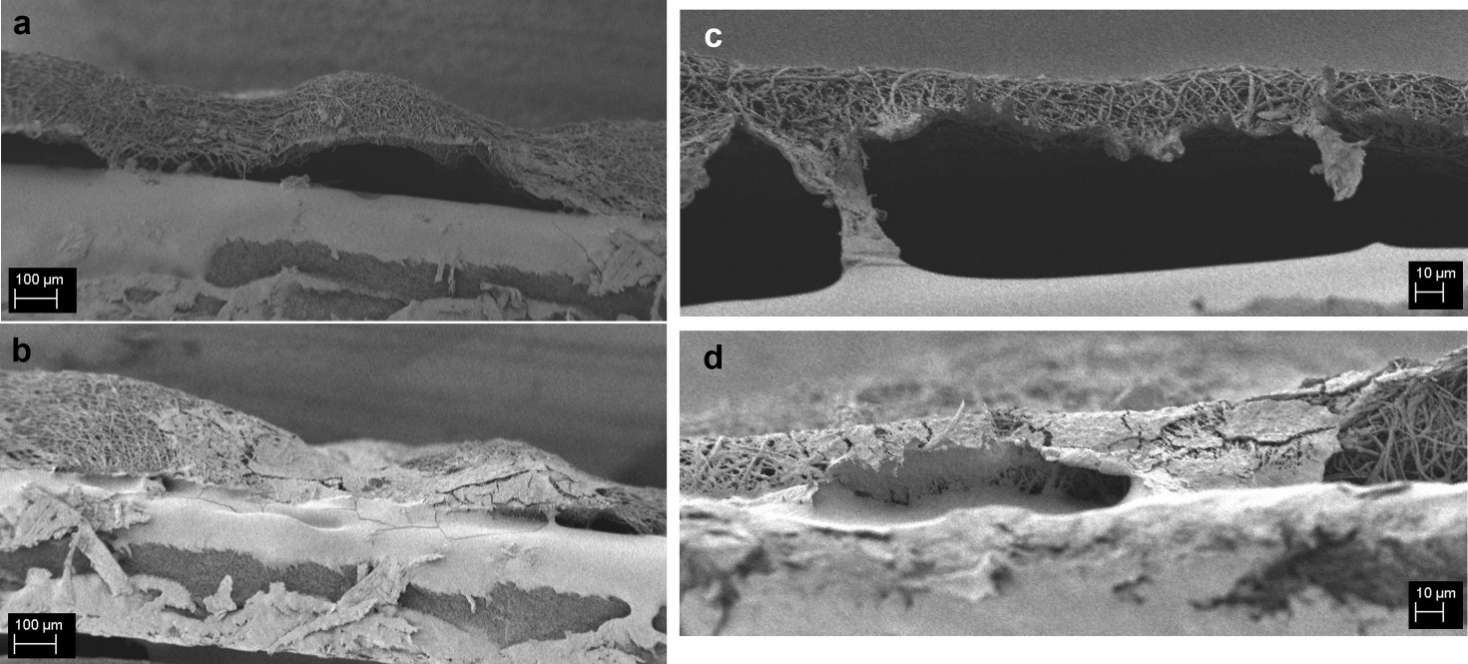
SEM micrographs (a,b) of PCL and (c,d) PCL/gelatin scaffolds without follicles (a,c) and with follicles (b,d) 10 days after the seeding (H&E staining). For PCL scaffolds, the magnification is 150X and the scale bar is 100 **μ**m for PCL/gelatin scaffolds the magnification is 1000X and the scale bar is 10 **μ**m.

The same sample stained with H&E was then sputtered and observed by SEM, results are reported in Fig 4B (PCL) and 4d (PCL/gelatin). Also neat PCL and PCL/gelatin blended scaffolds without follicles were mounted on the cross-section holders and used as control (Fig 4A and 4C). From this analysis it is possible to observe that this characterization method is effective to investigate the morphology of the fibers for both PCL and its blend in the cross-section as well as to obtain an accurate measurement of the scaffold thickness. In addition to this relevant information on the scaffold structure, since we are more interested in the characterization of the infiltration of the follicles inside the scaffolds, a more careful analysis of the seeded samples was performed and reported in Fig 4B and 4D.

For both seeded samples, it is possible to confirm that the scaffold fibrillary morphology was not altered after 10 days of culture in medium. The presence of the follicles in the section could be detected, in particular in comparison to the control samples. Besides the detection of their presence it is not possible to get more information about the follicles structure and also the typical spherical shape seems to be not preserved. The lack of preservation of the three-dimensional spherical shape could be ascribable to the drying process. In fact, after the staining with H&E the samples were dried at room temperature and therefore the loss of spherical morphology might have occurred. These considerations are valid for both types of scaffolds, confirming that this technique (H&E staining and SEM analysis) is not suitable for the assessment of the follicles infiltration.

**Fig 5:**
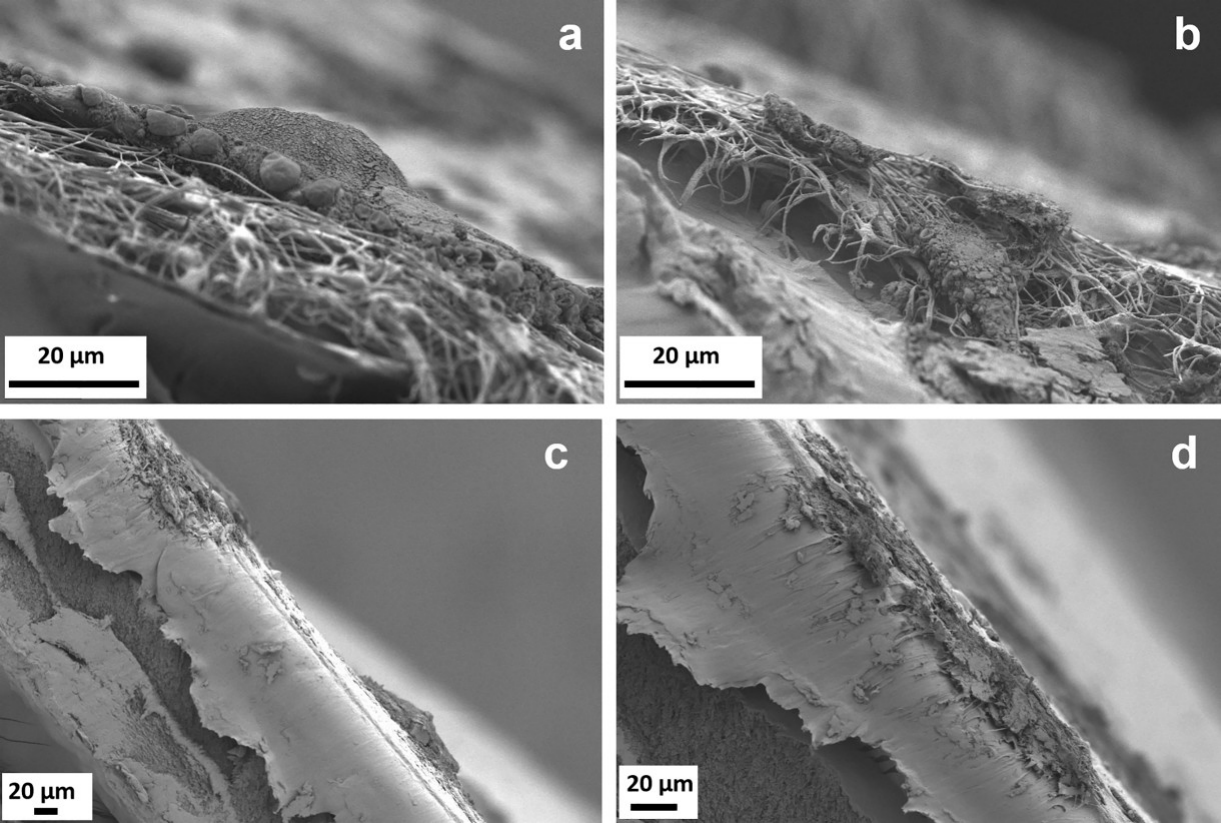
SEM micrographs of electrospun scaffolds with follicles 10 days after the seeding (a,b) PCL scaffold, magnification 3000X, scale bar 20 **μ**m and (c,d) PCL/gelatin scaffolds, at different magnifications: (c) 500X and (d) 1000X, scale bar 20 **μ**m.

In terms of the SEM evaluation of the seeded scaffolds without previous H&E staining, the results are reported in Fig 5 for the PCL scaffolds and PCL/gelatin blend. It is important to notice that in this case, it is possible to observe the typical spherical shape of the follicles in the micrographs at lower magnification on the top of the electrospun scaffolds in Fig 5A and Fig 5C. We demonstrate in this way that the drying process during the sample preparation is crucial for the assessment of follicles morphology, in particular the use of critical point drying is strongly advisable for the evaluation of the three-dimensional structure of the seeded samples. Nevertheless, in terms of the assessment of the follicles present on the cross-section, it is possible to notice that their structure is damaged and it is not possible to surely claim their infiltration inside the scaffolds structure.

As it is possible to notice, both analyzed methods, namely H&E (with light microscopy and SEM) and SEM, are not effective for the analysis of the follicles infiltration. In fact the ineffective cut of the samples induced rupture of the follicles structures and also the morphology of the scaffolds seems was altered.

**Fig 6:**
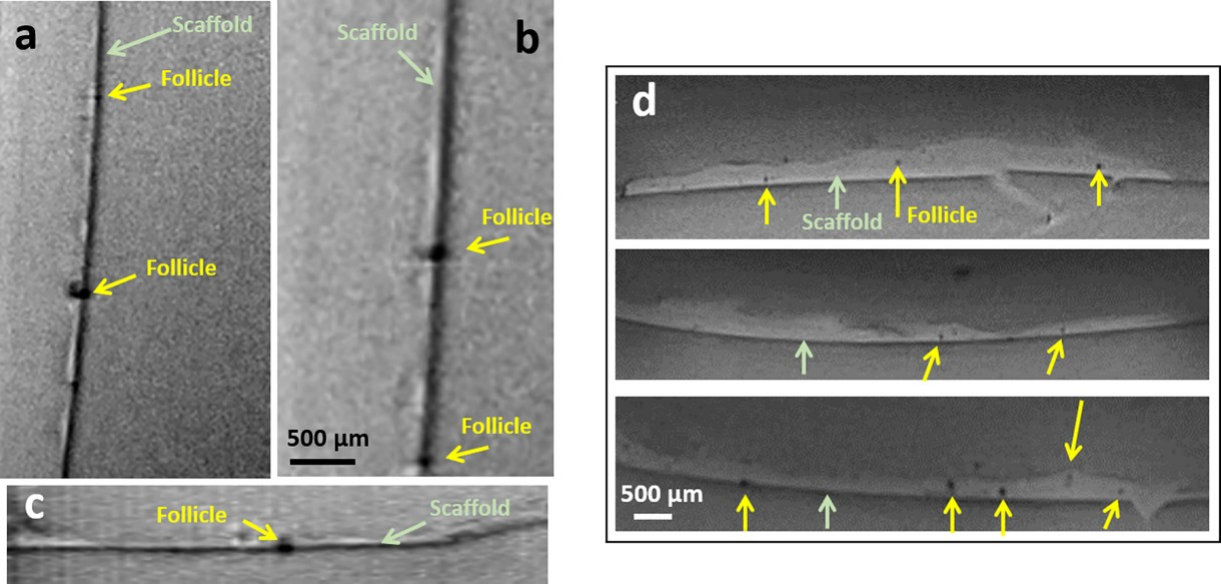
MR images of agarose embedded scaffolds with a resolution of 23x23x80**μ**m^3^ (a,b,c) PCL scaffold and (d) PCL/gelatin scaffold, scale bar 500 **μ**m.

With regard to the assessment performed with MRI, the first advantage is that this method is not destructive, indeed the seeded scaffolds were not cut during the sample preparation but just embedded in agarose, as reported in the scheme of the sample preparation (Fig 2), keeping the original morphology of the scaffolds and follicles, during the investigation of their section. Satisfactory results were obtained by the use of MRI to assess the follicles infiltration of both the scaffolds type (PCL and PCL/gelatin), as reported in Fig 6; associated MRI videos are provided as S1 and S2 Videos for PCL and PCL/gelatin, respectively. In Fig 6, reporting section of the seeded scaffolds, the scaffold structure is visible in light gray and it is indicated by green arrows, while the follicles, structures with low proton density, are dark and they are indicated by yellow arrows.

**Fig 7:**
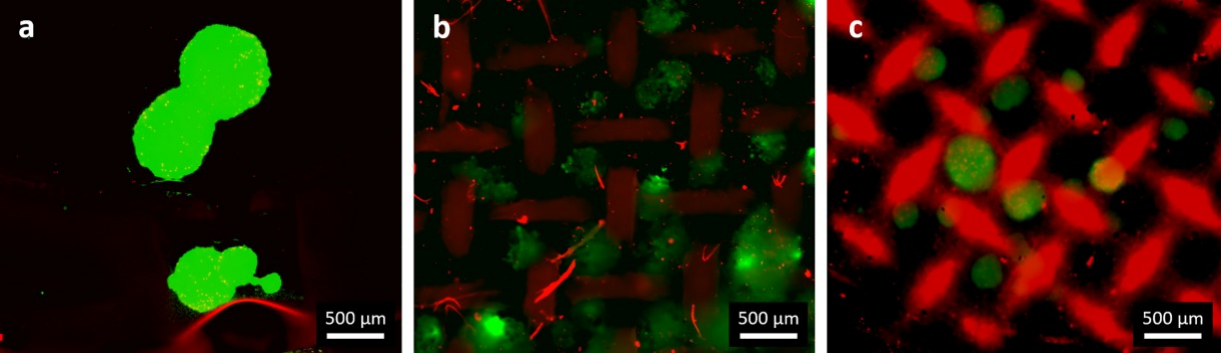
Representative fluorescence micrographs of LIVE/DEAD assay (a) control (PET membrane), (b) PCL and (c) PCL/gelatin scaffolds, magnification 2.5X, scale bar 500 μm.

In order to confirm the viability of the follicles inside the scaffolds 10 days after the seeding, LIVE/DEAD assay was performed and representative results are reported in Fig 7. The results confirmed that the follicles are viable in all the samples. In particular, for the electrospun scaffolds it is possible to observe the structure of the scaffold in red, in this way it is also clear to identify the position in which the follicles adhere. In fact, for PCL scaffolds the follicles are located mainly in the voids of the net, while in PCL/gelatin scaffolds the follicles seem to adhere on the scaffolds without following the pattern struts, as for PCL sample. This result confirms the relevance of the struts geometry as previously reported by Laronda et al. [8], but it adds also relevant information on the pivotal role played by the selected biomaterial.

**Fig 8:**
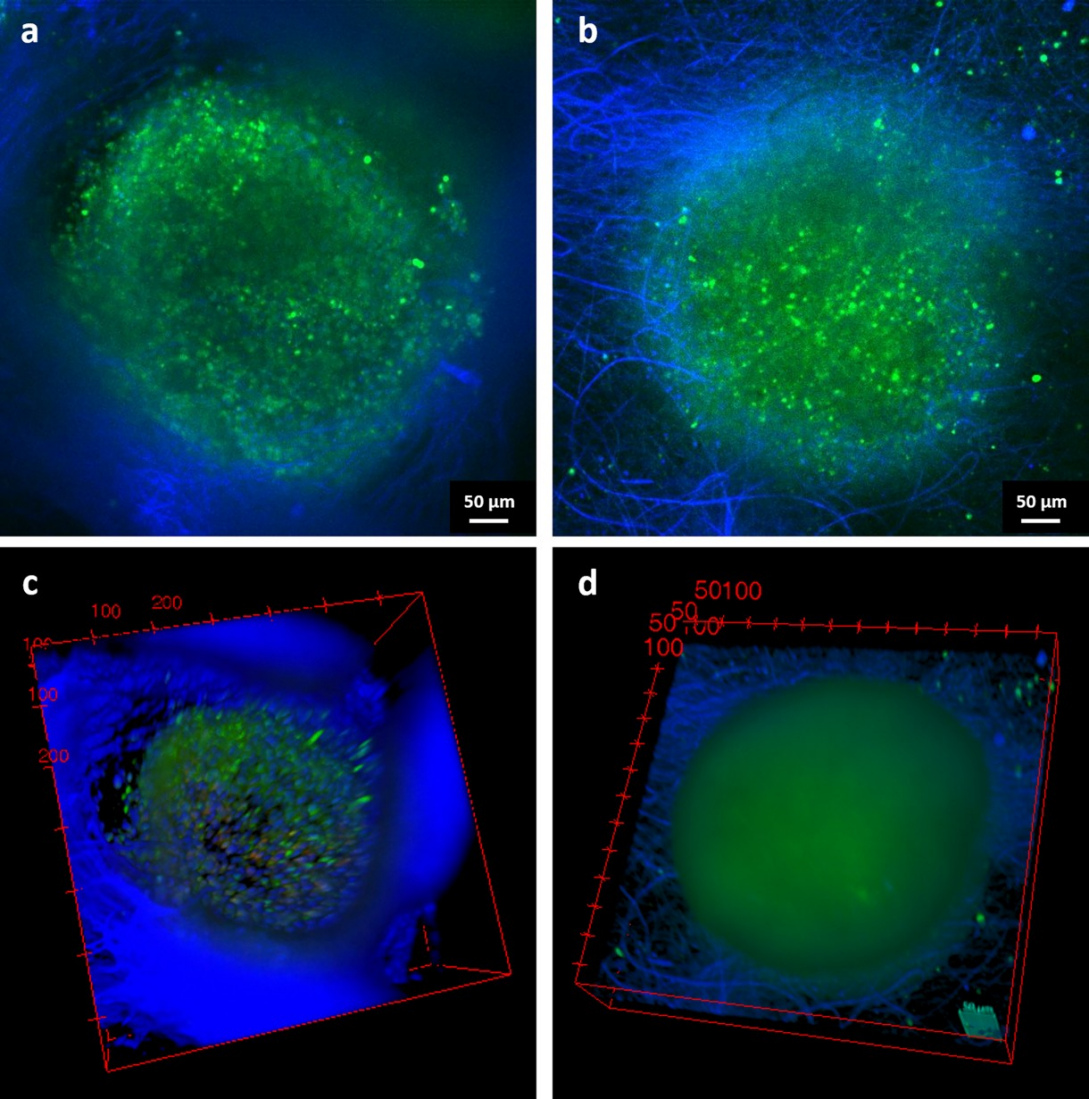
Confocal microscopy images of the follicles inside the scaffolds (a,c) PCL and (b,d) PCL/gelatin. Green points correspond to living cells, blue fibers represent the electrospun mesh and, within the follicle, the presence of single cells. Snapshot of 3D View are reported in (c,d). Scale bar 50 μm.

Confocal microscopy analysis was previously used by Laronda et al. [8] to assess the infiltration and adhesion of the follicles to the struts of the 3D printed scaffold. In the present work, living confocal imaging, reported as image stacks in Fig 8, was added as additional technique to investigate the infiltration of the follicles inside the scaffolds. For both PCL (Fig 8A and 8C) and PCL/gelatin (Fig 8B and 8D) the infiltration and the adhesion to the electrospun scaffolds is confirmed. In particular, from the videos of confocal analysis provided as S3 and S4 Videos from PCL and PCL/gelatin, respectively, it is possible to notice that the follicles are embedded and adherent to the scaffold structure. The same information is also reported in Fig 8C and 8D in which a snapshot of 3D Viewer (obtained by software) is reported. For PCL electrospun pattern, it is also possible to clearly observe that the follicle adheres inside the pore, confirming that the pattern does not inhibit or alter the adhesion of the follicles. The calcein staining (green) confirmed huge numbers of vital cells within the whole follicle in all images of the stack for both scaffold types and moreover, cells DNA and scaffold fibers are stained in blue.

As showed in the MRI videos in the Supplementary Information and corresponding images in Fig 6, the follicles are present not only on the surface of the scaffolds, but they infiltrate inside the electrospun fibrous scaffold, as also confirmed by confocal microscope analysis, reported in Fig 8. These results confirm the suitability of MRI technique to be used in REPRON applications for the evaluation of the infiltration of follicles in the scaffolds. From the obtained results, it is therefore also possible to highlight that a higher number of follicles could be detected inside PCL/gelatin scaffolds, confirming the relevance of the selection of proper biomaterials during the design process of the scaffolds.

The use of MRI for tissue engineering applications is still far to be considered a standard characterization technique for several reasons, i.e. the availability and cost related to MRI facilities, the need of experts for the analysis and the evaluation of the data, the lack of standardization, as already reported in literature [17]. Nevertheless, these first promising results suggest MRI as a method for effective analyzing of follicle infiltration inside electrospun scaffolds. Through development of a standardized protocol for the whole procedure, further advantages of MRI would become apparent, for example determining follicle diameters and volumes would be feasible by using the herein presented MRI of agarose embedded samples in combination with image processing tools.

## Conclusions

In conclusion, H&E staining with subsequent light microscopy or SEM analysis is not suitable for the evaluation of follicles infiltration in electrospun scaffolds; this is also true for SEM analysis alone. Nevertheless, SEM is effective to investigate fiber morphology as well as to precisely measure scaffold thickness; using critical point drying is strongly advisable, as it keeps the three-dimensional structure intact. Promising results were obtained by the use of MRI to assess follicles infiltration in the scaffolds. Precedent agarose embedding should be performed to fixate the specimens; this fixation technique further preserves the original morphology of scaffolds and follicles. In conclusion, the obtained results confirm that MRI is a promising technique with high perspectives for further applications in this field.

## Supporting information

S1 Video MR video of agarose embedded PCL scaffold seeded with ovarian follicles with a resolution of 23x23x80μm^3^. (MP4)

S2 Video MR video of agarose embedded PCL/gelatin scaffold seeded with ovarian follicles with a resolution of 23x23x80μm^3^. (MP4)

S3 Video Video from stacks of confocal images obtained with ImageJ 3D Viewer of PCL scaffold seeded with ovarian follicles. Calcein staining in green and in blue cells DNA and scaffold fibers.
(MP4)

S4 Video Video from stacks of confocal images obtained with ImageJ 3D Viewer of PCL/gelatin scaffold seeded with ovarian follicles. Calcein staining in green and in blue cells DNA and scaffold fibers.
(AVI)
